# Inferring population connectivity across the range of distribution of the stiletto shrimp *Artemesia longinaris* Spence Bate, 1888 (Decapoda, Penaeidae) from DNA barcoding: implications for fishery management

**DOI:** 10.3897/zookeys.457.6569

**Published:** 2014-11-25

**Authors:** Abner Carvalho-Batista, Mariana Negri, Leonardo G. Pileggi, Antonio L. Castilho, Rogério C. Costa, Fernando L. Mantelatto

**Affiliations:** 1 Laboratory of Biology of Marine and Fresh Water Shrimps, Faculty of Science, Department of Biological Sciences, São Paulo State University (UNESP), Bauru, São Paulo, Brazil; 2 Laboratory of Bioecology and Crustacean Systematics, Faculty of Philosophy, Sciences and Letters at Ribeirão Preto (FFCLRP), University of São Paulo (USP), Postgraduate Program in Comparative Biology, Ribeirão Preto, São Paulo, Brazil; 3 São Paulo State University (UNESP), Biosciences Institute of Botucatu, Zoology Department, Botucatu, Brazil

**Keywords:** Cytochrome Oxidase I, gene flow, Penaeoidea, phenotypic plasticity

## Abstract

*Artemesia longinaris* is a marine shrimp endemic to the southwestern Atlantic and distributed from Atafona, Rio de Janeiro (Brazil) to Rawson, Chubut (Argentina). In recent years, this species has become an important target of the commercial fishery as a consequence of the decline in the fishery of more traditional and profitable marine shrimps. In addition, phenotypic variations have been documented in populations along its distribution. Therefore, investigations on the genetics of the fishing stocks are necessary for the development of sustainable management strategies and for understanding the possible sources of these variations. The mitochondrial gene Cytochrome Oxidase I (COI) was used to search for evidence of genetic structure among the populations of *Artemesia longinaris* and to analyze the phylogenetic relationships among them. A total of 60 specimens were collected from seven different localities, covering its geographical range. The final alignment showed 53 haplotypes (48 individuals and 5 shared), with no biogeographical pattern. The low genetic divergence found, with a non-significant FST value, also suggests the absence of population structure for this gene. These findings indicate a continuous gene flow among the populations analyzed, suggesting that the phenotypic variation is a consequence of different environmental conditions among the localities.

## Introduction

*Artemesia longinaris* Spence Bate, popularly known as Argentine stiletto shrimp, plays an important role in the marine trophic chain of the southwestern Atlantic, as food for different species of fish and cephalopods (Capitoli et al. 1994). In recent years, however, this species has become a common target of both artisanal and industrial fisheries. The former occurs along its entire distribution and the latter is mainly concentrated in southern Brazil and Argentina (D’Incao et al. 2002). The increase in the fishery of *Artemesia longinaris* is a consequence of a decline in the stocks of more traditional and profitable marine shrimps, such as the pink shrimp *Farfantepenaeus brasiliensis* (Latreille) and *Farfantepenaeus paulensis* (Pérez-Farfante), the white shrimp *Litopenaeus schmitti* (Burkenroad) and the seabob shrimp *Xiphopenaeus kroyeri* (Heller) (D’Incao et al. 2002, Costa et al. 2004, Carvalho-Batista et al. 2011).

In the last decades, catches in the states of south and southeast Brazil have reached thousands of tons (D’Incao et al. 2002). Furthermore, in spite of the increase in its exploitation in recent years, there is no specific management plan for *Artemesia longinaris* in Brazil. The offseason in south and southeast coast of this country for this species and other commercial shrimps is based on the period of juvenile recruitment of *Farfantepenaeus* species, without taking account the possibility of the existence of more than one stock for these species (Franco et al. 2009).

*Artemesia longinaris* has a distribution restricted to the southwestern Atlantic, from Atafona (Rio de Janeiro, Brazil, 21°37'S) to Rawson (Chubut, Argentina, 43°18'S) (D´Incao 1999). Although its distribution is limited to the Argentinean biogeographical province, much of its extent (23° to 35°S) is considered a transitional region because of current mixing; this process leads to the formation of water masses with tropical and subantarctic characteristics (Boschi 2000). In addition, the northern boundary of its distribution is located in the region of Cabo Frio (Rio de Janeiro, Brazil), where there is a strong influence of upwelling events, driven by the winds and coastal topography (Acha et al. 2004).

Consequently, environmental conditions differ considerably throughout the range of *Artemesia longinaris*. For example, in the Ubatuba region (São Paulo, Brazil) the temperature (16–30 °C) and salinity (28–38) vary widely because of the intrusion of different water masses (Fransozo et al. 2004, Costa et al. 2005); whereas near Cabo Frio (Rio de Janeiro, Brazil) the water temperature is about 20 °C and the salinity is high (>37) during most of the year (Sancinetti 2011); and on Mar del Plata coast (Buenos Aires, Argentina) the temperature varies seasonally, from 6 to 17 °C, and the salinity is slightly greater than 30 (Petriella and Bridi 1992, Guerrero et al. 1997, Acha et al. 2003).

In addition, phenotypic variations among *Artemesia longinaris* populations have been noted. The body size and the mean size at sexual maturity (CL_50%_) increase with the latitude, from Ubatuba (São Paulo, Brazil) to Mar del Plata (Buenos Aires, Argentina), but decrease with latitude from the Farol de São Tome (Rio de Janeiro, Brazil) to Ubatuba (Boschi 1969a, Ruffino and Castello 1992, Castilho et al. 2007b, Semensato and Di Beneditto 2008, Costa et al. 2010). Differences in certain morphometric relationships have also been detected (Dumont and D’Incao 2010), as well as in the reproductive period, which tends to be continuous in lower latitudes and seasonal in higher latitudes (Christiansen and Scelzo 1971, Petriella and Bridi 1992, Castilho et al. 2007a).

In view of these environmental variations, Nascimento (1983) proposed that the populations off southern Brazil and northern Argentina are likely separated, based on the differences in their environmental preferences. However, an analysis of enzyme polymorphisms provided no support for this proposition (Weber et al. 1993). Further studies to investigate the possibility of genetic structure and covering the entire distribution of *Artemesia longinaris* were still lacking.

Knowledge of the genetic structure of populations is important for the development and success of strategies for sustainable long-term management of fishery resources (Hillis et al. 1996). Mitochondrial DNA has been an important tool for these investigations, for terrestrial as well as aquatic organisms (Avise 1994). Among the mitochondrial molecular markers, the Cytochrome Oxidase I (COI) gene has been successfully employed to detect population structures in many species of Decapoda (Schubart and Huber 2006, Aoki et al. 2012, De Croos and Pálsoon 2012, Terossi and Mantelatto 2012). This property, together with other characteristics, has resulted in the choice of this gene as the standard marker for animal identification in the DNA barcoding technique (Hebert et al. 2003).

This study had the following aims: to evaluate the hypothesis of genetic structure among the populations of *Artemesia longinaris*; investigate their phylogenetic relationships; and detect, if possible, evidences of speciation. To achieve these purposes, we used a partial sequence of the mitochondrial COI gene as the molecular marker. The population concept adopted was proposed by Roughgarden et al. (1989) and Krebs (1994). According to them, a population is a group of organisms of the same species that occupy the same place at a certain time. Our findings provide an appropriate theoretical basis for the development of management strategies for this fishery resource, as well as help to understand the origin of the phenotypic differences among populations of this species.

## Methods

### Sample collection

**Figure 1.**
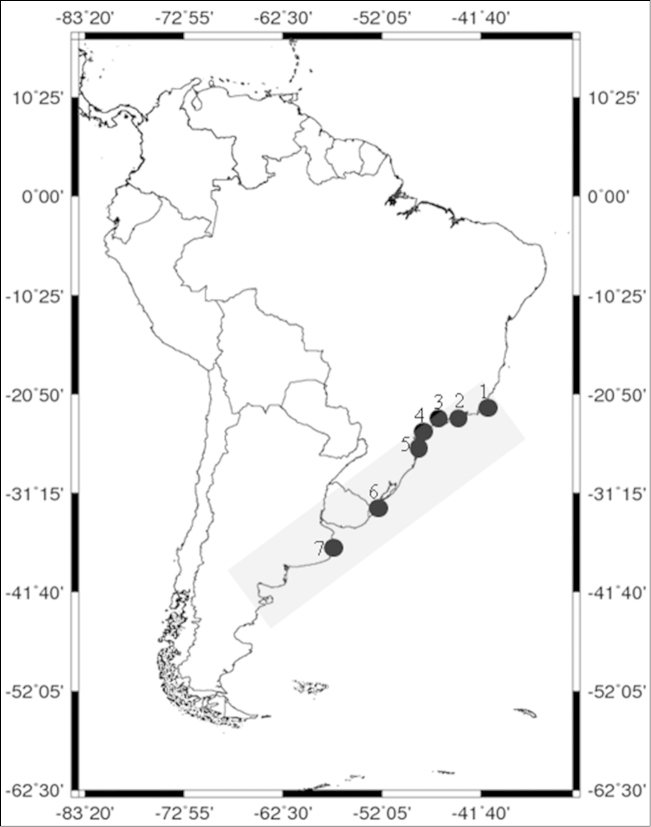
Southwest Atlantic collection sites. Map showing the localities of the specimens of *Artemesia longinaris* analyzed: **1** Macaé, Brazil **2** Ubatuba, Brazil **3** Santos, Brazil **4** Cananéia, Brazil **5** São Francisco do Sul, Brazil **6** Rio Grande, Brazil **7** Mar del Plata, Argentina. The gray band indicates the complete geographical distribution of *Artemesia longinaris*.

The specimens were obtained, at scientific cruises, from seven localities in the southwestern Atlantic (Table 1 and Fig. 1). The specimens were identified based on Costa et al. (2003), and were immediately preserved in 80% ethanol and deposited in the Crustacean Collection of the Department of Biology (CCDB), Faculty of Philosophy, Sciences and Letters at Ribeirão Preto (FFCLRP), University of São Paulo (USP) (Table 1).

**Table 1.** List of specimens used for molecular analysis with respective site of collection, catalogue numbers, and GenBank accession numbers of *Artemesia longinaris*. The letters CCDB preceding the catalogue numbers represent the Crustacean Collection of the Department of Biology, Faculty of Philosophy, Sciences and Letters at Ribeirão Preto, University of São Paulo.

Locality	Catalogue numbers	GenBank Accession Numbers
Macaé-Rio de Janeiro, Brazil (22°23'44"S; 41°44'57"W)	CCDB 3782	KF572060–KF572069
Ubatuba-São Paulo, Brazil (23°27'24"S; 45°01'20"W)	CCDB 3806, 3429	KF572070–KF572082
Santos-São Paulo, Brazil (24°03'59"S; 46°16'57"W)	CCDB 4008	KF572083–KF572084
Cananéia-São Paulo, Brazil (25°08'15"S; 47°50'40"W)	CCDB 3655	KF572085–KF572089
São Francisco do Sul-Santa Catarina, Brazil (26°05'52"S; 48°33'82"W)	CCDB 3851	KF572090–KF572098
Rio Grande-Rio Grande do Sul, Brazil (32°10'23"S; 52°06'10"W)	CCDB 3928	KF572099–KF572108
Mar del Plata-Buenos Aires, Argentina (37°58'57"S; 57°32'15"W)	CCDB 869, 4150	KF572109–KF572119

### DNA extraction, PCR amplification, purification and sequencing

The protocols for DNA extraction, amplification and sequencing followed Mantelatto et al. (2009) and Pileggi and Mantelatto (2010).

An ~700-bp region of a partial sequence of the mitochondrial COI gene was amplified by the polymerase chain reaction (PCR) using the pair of primers: HCO1 (5’-TAAACTTCAGGGTGACCAAAAAATCA-3’) and LCO1 (5’-GGTCAACAAATCATAAAGATATTGG-3’) (Folmer et al. 1994). The PCR reaction was performed in an Applied Biosystems Veriti® 96-well thermocycler, using the following thermal cycle: initial denaturing for 2 min at 94 °C followed by 35 denaturing cycles at 94 °C for 30 s, primer annealing at 50–58 °C for 30 s and extension at 72 °C for 1 min, and a final extension for 5 min at 72 °C. The PCR products were purified using the SureClean Plus® purification kit (Bioline) and were sequenced with the Big Dye® Terminator Cycle Sequencing kit in an ABI 3100 Genetic Analyzer® (Applied Biosystems Life Technologies). All sequences were conﬁrmed by sequencing both strands.

### Data analysis

The editing and construction of a consensus sequence for the two strands were conducted using the computational program BIOEDIT 7.3.1.0 (Hall 1999). Sequences were aligned using the program CLUSTAL W (Thompson et al. 1994), with interface to BIOEDIT (Hall 1999) using default parameters. The computational program MEGA 5.0 (Tamura et al. 2011) was used to estimate the average nucleotide composition and genetic distances, and to construct a Neighbor-Joining dendrogram, both based on the Kimura 2-parameter substitution model (Kimura 1980). The phylogram using the Maximum Likelihood criterion was constructed in the program RAxML-HPC2 on X-SEDE (Stamatakis 2006) through the online version of the Cyber Infrastructure for Phylogenetic Research (CIPRES) website (Stamatakis et al. 2008, Miller et al. 2010). The default parameters of RAxML were used to perform the analysis for the GTR model. To measure the consistency of the topology, we selected the option to automatically determine the number of bootstraps to be run in RAxML. Consequently, 1000 bootstrap pseudo-replicates were run, and only the values >50% were reported.

For both the genetic distance and phylogenetic analyses, sequences of three other penaeid species were included in the alignment as an outgroup: *Farfantepenaeus brasiliensis*, *Farfantepenaeus paulensis* (GenBank accession numbers KF783861–KF783862) and *Rimapenaeus constrictus* (Stimpson) (GenBank accession number KF783863). We also attempted to use a sequence of the same portion of the COI gene of *Artemesia longinaris* available in GenBank (accession number EU400383.1) (Dumont et al. 2009). However, it was not possible to obtain alignments without gaps when this sequence was included. This observation, allied to the fact that its translation to an amino-acid sequence showed the presence of stop codons, indicates that this sequence must be reviewed. The presence of stop codons in the middle of an encoding gene suggests the possibility of the amplification and sequencing of a pseudogene (Buhay 2009).

The haplotype number was calculated in the program DNASP 4.10.9 (Rozas and Rozas 1999). The haplotype network was constructed by the Median-Joining method in NETWORK software (Bandelt et al. 1999), with data preparation in DNASP. The haplotype and nucleotide diversities were calculated for each locality using ARLEQUIN Version 3.1 (Excoffier et al. 2005). The genetic variation was analyzed with a analysis of molecular variance (AMOVA) (Excoffier et al. 1992), and was computed in ARLEQUIN Version 3.1 (Excoffier et al. 2005).

## Results

A total of 60 sequences of the COI gene from individuals sampled in the seven localities was obtained. The final multiple sequence alignment included 645 base pairs. The number of variable sites was 66 (10.23%), 8 (12.12%) in the first codon position and 58 (87.88%) in the third position, and 30 of the variable sites were phylogenetically informative. Adding three species as the outgroup, the number of variable sites was 143 (28.49%), 72 of which were phylogenetically informative. The average nucleotide composition for *Artemesia longinaris* was 28.41% (A), 30.99% (T), 19.47% (G), and 21.12% (C).

The intraspecific genetic distance of *Artemesia longinaris* ranged from 0 to 2.7%, and the average distance was 1.1 ± 0.2%. The interspecific genetic distance, including the outgroup, ranged from 21.3 to 27.1%. Average distance among individuals in each population ranged from 0.81 ± 0.25% at Cananéia to 1.42 ± 0.24% at Macaé (Table 2). Among localities, distances ranged from 0.8 ± 0.2% between Santos and Cananéia to 1.4 ± 0.2% between Macaé and São Francisco do Sul (Table 3).

**Table 2.** Average distance (%) among *Artemesia longinaris* individuals ± standard deviation in each locality.

Locality	Average distance (%)	Standard deviation (±)
Macaé	1.42	0.24
Ubatuba	1.07	0.19
Santos	1.25	0.43
Cananéia	0.81	0.25
São Francisco do Sul	1.37	0.26
Rio Grande	1.08	0.19
Mar del Plata	0.88	0.21

**Table 3.** *Artemesia longinaris*Average distance (%) among localities (numbers on bottom) ± standard deviation (values on top).

Locality	1	2	3	4	5	6	7
**1 Macaé**		0.19	0.25	0.21	0.22	0.19	0.19
**2 Ubatuba**	1.21		0.23	0.18	0.19	0.18	0.17
**3 Santos**	1.30	1.08		0.23	0.23	0.23	0.23
**4 Cananéia**	1.17	0.95	0.78		0.20	0.18	0.19
**5 São Francisco do Sul**	1.37	1.21	1.13	1.02		0.19	0.20
**6 Rio Grande**	1.20	1.04	1.13	0.96	1.20		0.17
**7 Mar del Plata**	1.16	0.96	0.97	0.83	1.11	0.95	

**Figure 2.**
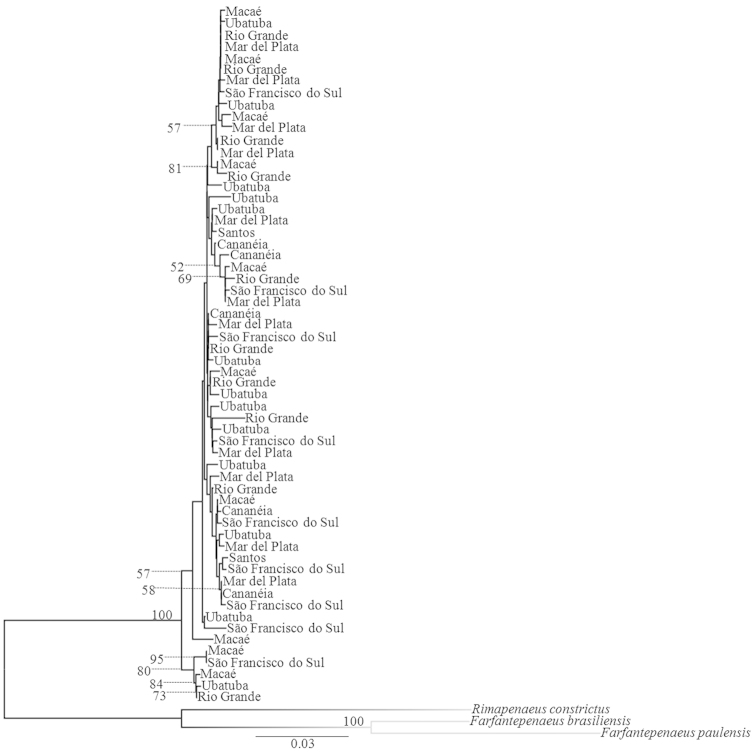
Dendrogram based on Neighbor-Joining distance method of COI gene sequences of individuals of *Artemesia longinaris*. Localities represent the analyzed specimens. Numbers are bootstrap support values (1000 replicates); values below 50% are not shown.

**Figure 3.**
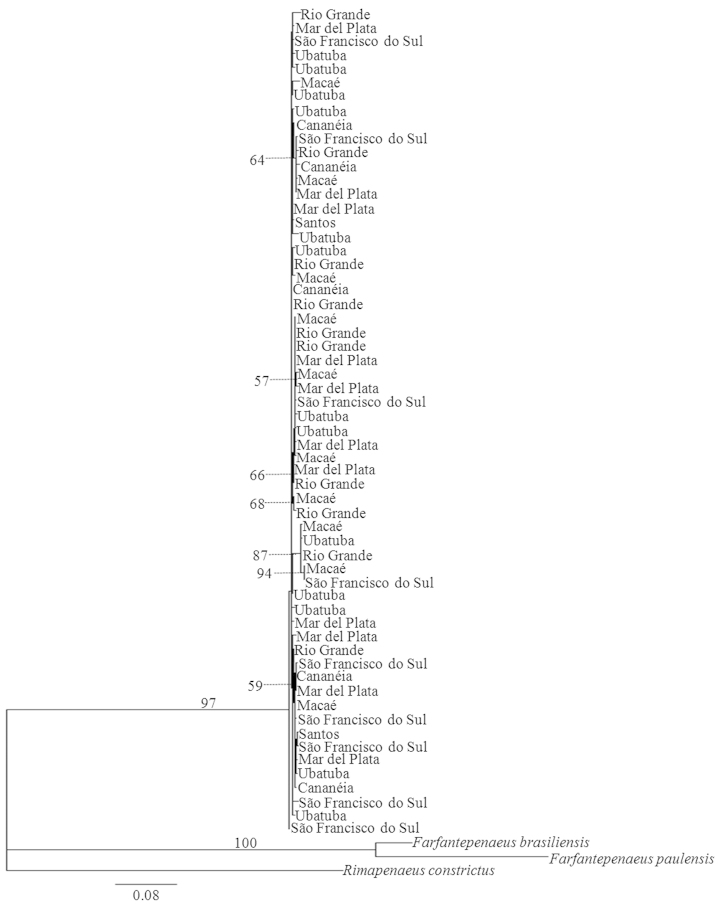
Phylogram for individuals of *Artemesia longinaris* inferred from Maximum Likelihood analysis of COI gene sequences. Localities represent analyzed specimens. Numbers are bootstrap support values (1000 replicates); values below 50% are not shown.

Both the Neighbor-Joining and Maximum Likelihood analysis indicated no structure by localities (Figs 2 and 3).

**Figure 4.**
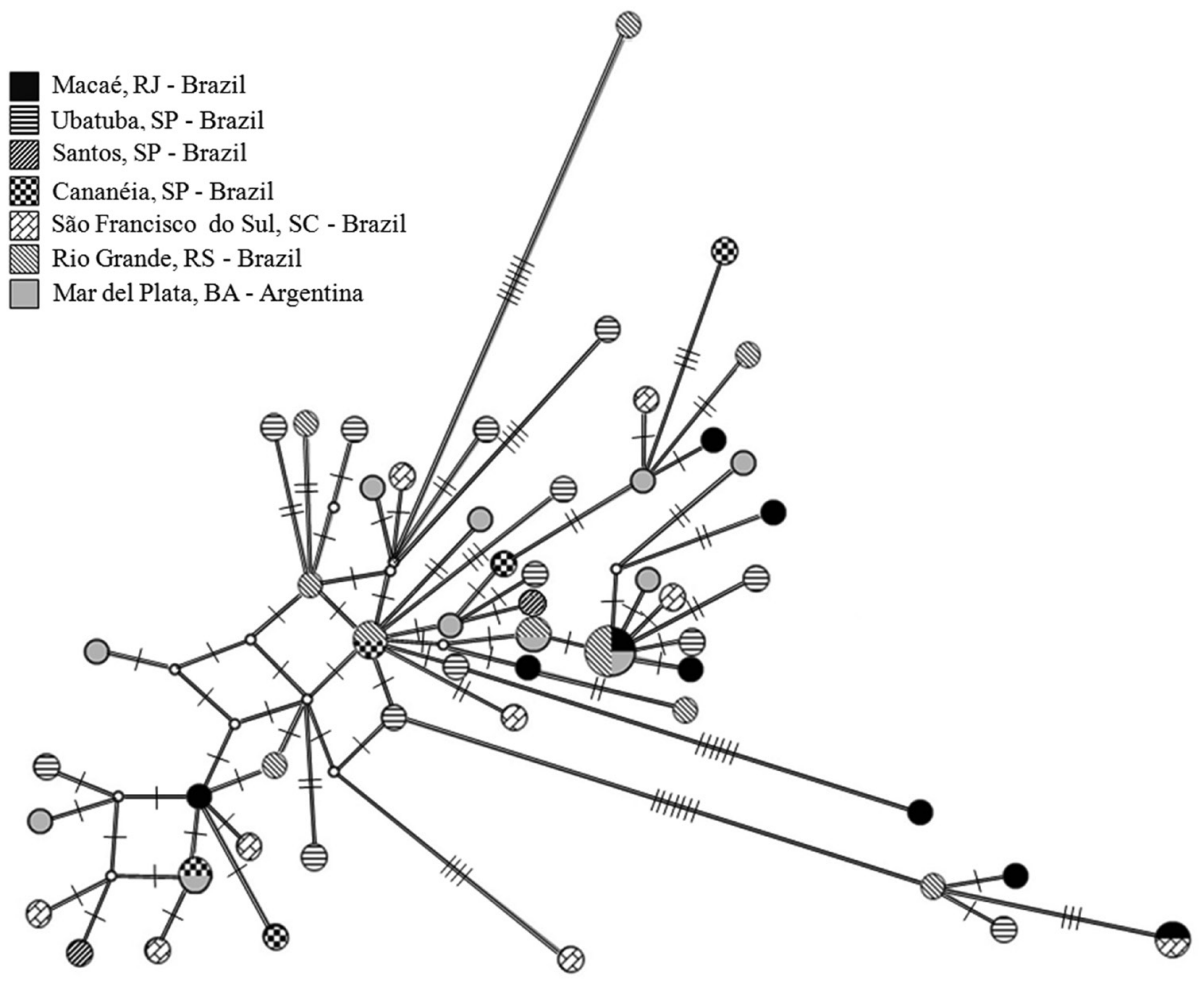
Haplotype network of *Artemesia longinaris*according to Median-Joining analysis. Each circle represent one haplotype found in the localities (53 haplotypes in 60 specimens). The size of the circle of each haplotype is proportional to its frequency in the sample. Each small dash represents a mutational step.

Based on the 60 sequences, 53 haplotypes were identified. Of these, 48 represented single individuals. The locality of Santos was not included in the analysis of haplotype, nucleotide diversity and molecular variance (Tables 4 and 5), since only two sequences were obtained from this site. The caught of *Artemesia longinaris* in this locality is difficult, occurring only in some occasions with low temperatures and often in low abundances (Carvalho-Batista et al. 2011). The haplotype network did not reveal any genetic structure among groups (Fig. 4). Five haplotypes were shared, and the most frequent one was observed in four specimens from three localities (Fig. 4).

**Table 4.** Number of *Artemesia longinaris* individuals sampled, number of haplotypes, D.H. = haplotype diversity, and D.N. ± D.P. = nucleotide diversity ± standard deviation for each locality.

Locality	Number of samples	Number of haplotypes	D. H.	D. N. ± D. P.
Macaé	10	10	0.10	1.38×10^-3^ ± 0.79×10^-3^
Ubatuba	13	13	0.08	1.05×10^-3^ ± 0.4×10^-3^
Santos	2	2		
Cananéia	5	5	0.20	0.80×10^-3^ ± 0.5×10^-3^
São Francisco do Sul	9	9	0.11	1.34×10^-3^ ± 0.8×10^-3^
Rio Grande	10	9	0.12	1.05×10^-3^ ± 0.6×10^-3^
Mar del Plata	11	11	0.91	0.87×10^-3^ ± 0.5×10^-3^

The analysis of molecular variance (AMOVA) did not detect structure among the localities, and the observed variation occurred predominantly within the localities. The FST indices were not significant (p > 0.05) (Table 5).

**Table 5.** Analysis of molecular variance (AMOVA) performed with specimens of *Artemesia longinaris* obtained from seven localities. *Significant values, *P* < 0.05.

Structure	Variation Source	%	Fixation index	P
Absent	Among localities	-1.80	FST: -0.02	0.95
	Within localities	101.80		

## Discussion

The intraspecific genetic distance for *Artemesia longinaris* (0–2.7%) is much lower than the interspecific distance between *Artemesia longinaris* and the out-group species (21.3–27.1%). This result not only confirms *Artemesia longinaris* as a single taxon throughout its distribution, but also supports the utilization of this methodology in the identification of penaeid shrimps from the Brazilian coast. The difference between the intra and interspecific genetic variation of the barcode region of the COI gene is termed the “barcode gap” (Hebert et al. 2004). It is an efficient method for differentiating species through the DNA Barcoding technique (Hebert et al. 2004, Waugh 2007, Frézal and Leblois 2008, Ward 2009). The genetic divergence values are consistent with other studies involving the family Penaeidae, with intraspecific values lower than 3.5% and interspecific values generally higher than 10% (in some cases exceeding 20%) (Gao et al. 2003, Quan et al. 2004, Keskin and Atar 2013).

Our analyses showed genetic homogeneity among the populations of *Artemesia longinaris* along its entire geographical distribution. The FST value obtained reflects this absence of geographical genetic structure. In species with high genetic variation and few shared haplotypes, negative FST values are probably associated with the imprecision of the algorithms used in this type of analysis, and can be interpreted as zero (Winkelmann et al. 2013).

Despite the absence of significant genetic variability at the intraspecific level described here, phenotypic variability was previously observed among the populations of *Artemesia longinaris* (see introduction). The determination of an individual phenotype is a consequence of the interaction between genotype and environment (Templeton 2006). Thus, the same genotype may be associated with different phenotypes under different environmental conditions (Miner et al. 2005, Vogt et al. 2008, Sotka 2012).

Recent studies with other decapods, with sampling at several points of the South American coast, found similar results on genetic homogeneity (Laurenzano et al. 2012, Terossi and Mantelatto 2012, Rossi and Mantelatto 2013, Wieman et al. 2013, Laurenzano et al. 2013). These authors indicated the high capacity of planktonic larval dispersal as the main factor responsible for this homogeneity over their distributions, making it impossible to establish a population structure over this broad geographical range (Gopurenko and Hughes 2002).

We can conjecture that similar larval dispersal occurs with *Artemesia longinaris*, in which its larval development lasts 24 to 32 days, according to the temperature (Boschi and Scelzo 1977). This period is sufficient for the larvae to be passively transported for hundreds of kilometers by the currents (Palumbi 2003). The ability of larvae to travel for long distances was demonstrated for other penaeid shrimps. For example, larvae of *Pleoticus muelleri* (Spence Bate), on the Argentine coast, are able to travel for distances between 120 and 300 nautical miles (about 220 and 550 km, respectively), transported by the coastal currents (Boschi 1989).

It is thought that the dynamics of water masses in the region provides ideal conditions for larval drift of *Artemesia longinaris* through the southwestern Atlantic. Coastal Water (CW), for instance, is a water mass that cover the geographical range of this study (Campos et al. 2000), and can flow towards north or south depending on the wind conditions and season showing different properties of temperature and salinity, depending the region and the influence of other water masses (Piola et al. 2005, Calado et al. 2006, Castro-Filho et al. 2008) allowing larval dispersal to different areas.

According to Fransozo et al. (2004), Costa et al. (2005) and Carvalho-Batista et al. (2011), the occurrence of adults of *Artemesia longinaris* in São Paulo State is associated with the temperature decrease to 17–21 °C. During the spring (October to December) in Ubatuba, the number of animals in the larger size classes increased. It was associated with the coming of migrants into the population (Castilho et al. 2007a). Thus, the gene flow of *Artemesia longinaris* is not limited to larval drift, but also is a consequence of juvenile and adult migration. Penaeid migration over long distances was also evidenced by Ruello (1975), who recaptured a female of *Melicertus plebejus* (Hess), on the Australian coast, 930 km from the site where the specimen was marked.

Our results, encompassing samples from its entire distribution, support the hypothesis that *Artemesia longinaris* migrates over long distances, and is able to establish populations in different areas when conditions are appropriate. It is therefore possible to consider *Artemesia longinaris* as a metapopulation, which fits the model of source and sink proposed by Pulliam (1988). The populations (or subpopulations) that are continuously more stable and in high density throughout the year, such as those from southern Brazil to Argentina and from Macaé (Boschi 1969a, b, Nascimento 1983, Sancinetti 2011), are probably sources of new individuals for the less-stable populations, the sinks, such as the populations (or subpopulations) from São Paulo State.

Thus, these localities, where the populations are considered sources, would be strategic for the implementation of management measures such as the creation of protected areas or offseason periods, in order to maintain the fisheries in these areas and also in all range of its distribution. The role of marine protected areas in enhance fisheries in adjacent regions depend if they act as sources or as sinks (King 1995). Even connected one to each other, each subpopulation has its own dynamic (Begon et al. 2006), so the conservation policies must take into account the particular characteristics of each locality.

Studies investigating the larval dispersal and the migration of juveniles and adults of this species must be conducted in order to verify whether the model described by Pulliam (1988) is applicable or not. Apart from this, with the intent of providing a better quantification of the degree of exchange among the populations, as well as to evaluate the possibility of recent divergence among them, which is not detectable by the marker used here, additional molecular investigations using different genes are encouraged.

## Conclusion

Our results confirm that the DNA barcoding technique is an efficient tool for the identification of penaeid shrimps from the Brazilian coast. In addition to the validation of *Artemesia longinaris* as a single taxon, with no genetic differentiation among the populations through its entire geographical distribution, we showed the importance of the effect of the environmental conditions specific to each locality in the expression of the phenotypic characteristics of the individuals in a population.

The genetic homogeneity is maintained by the larval dispersal and high migratory capacity, which assure gene flow among populations. These characteristics make it possible for individuals to be transported by water masses and currents of the southwestern Atlantic.

In addition, this study also indicate the importance of populations of south Brazil and Macaé as sources, to provide individuals to other areas. Thus these populations should be considered essential in developing management strategies for the species.
